# Effects of enzyme preparations on growth performance, meat quality, and hepatic transcriptome of yaks

**DOI:** 10.3389/fvets.2026.1707381

**Published:** 2026-02-06

**Authors:** Yongfu La, Xiaoming Ma, Runli Wang, Futao Mo, Minghui Zhao, Yan Yang, Xuemei Liu, Maji Wan, Pengjia Bao, Min Chu, Xian Guo, Ping Yan, Chunnian Liang

**Affiliations:** 1 Key Laboratory of Animal Genetics and Breeding on Tibetan Plateau, Key Laboratory of Yak Breeding Engineering, Ministry of Agriculture and Rural Affairs, Lanzhou Institute of Husbandry and Pharmaceutical Sciences, Chinese Academy of Agricultural Sciences, Lanzhou, China; 2 Institute of Western Agriculture, The Chinese Academy of Agricultural Sciences, Changji, China; 3 Animal Husbandry Workstation of Hezuo, Hezuo, China; 4 Gansu Grassland Technical Extension Station, Lanzhou, China; 5 Qilian County Livestock and Veterinary Station, Qilian, China

**Keywords:** fatty acid composition, gene expression, meat quality, metabolomics, yak

## Abstract

The balance between grass and livestock has exacerbated the problem of high-quality development in the yak industry, and the nutritional regulation of enzyme preparations has become a research hotspot. The purpose of this study was to assess and contrast the effects of feeding with various concentrations of enzyme preparations of yaks, including the production performance, meat quality, fatty acid composition of the longissimus dorsi muscle, and gene expression. Adding 0.20% enzyme preparation to the diet can significantly increase the average daily gain (ADG), and reduce the dry matter intake (DMI) and feed conversion rate (FCR), but has no significant effect on the meat quality of yak. In addition, the liver transcriptome and muscle metabolize showed that the addition of enzyme preparations caused differential expression of fatty acid metabolism-related genes in the liver, which may affect the metabolism of fatty acids in the liver and thus alter the fatty acid composition in the longissimus dorsi muscle of yak. The results of this study emphasize that adding enzyme preparations can improve the production performance of yaks without affecting their meat quality, thereby enhancing the economic benefits of yak breeding.

## Introduction

Yaks play an indispensable role in the daily lives of Tibetan Plateau inhabitants, serving as a cornerstone of highland pastoralism (1). These animals provide vital resources such as milk, meat, fiber, and dung for fuel, while their strength and endurance make them essential for transportation across the region’s vast and rugged terrain (2–4). Adapted over millennia to extreme high altitude conditions including temperatures near freezing, hypoxic environments, and scarce forage yaks thrive where few other livestock can survive (5–7). However, growing demand for yak-derived products has led to intensified rearing practices, exacerbating pressure on already limited forage resources. This overreliance on yak herd risks ecological degradation, threatening both the plateau’s fragile ecosystems and the yaks’ own welfare. To address these challenges, modern technologies must be employed to reduce feed costs, enhance breeding efficiency, and improve economic returns all while ensuring sustainable environmental practices. Such measures are critical to safeguarding the region’s ecological balance and the long-term viability of yak husbandry.

Exogenous feed enzymes (EFEs) are supplemented into cattle diets to enhance nutrient utilization and overall animal performance (8). Early research in ruminant nutrition primarily focused on fibrolytic enzymes to improve fiber degradation (9). These studies demonstrated promising outcomes, particularly in increasing the average daily gain of cattle fed high-forage diets during the backgrounding phase (42). The benefits of EFEs are attributed to several mechanisms, such as (1) enhanced fiber hydrolysis in the preingestive, ruminal, and postruminal phases; (2) improved feed utilization through increased digestion and degradation rates; and (3) modulation of rumen dynamics. Specific modes of action may include accelerating ruminal passage rates, promoting microbial colonization of plant biomass, stimulating rumen microbial activity, and reducing digestive fluid viscosity (8). However, the efficacy of EFEs is highly variable and influenced by multiple factors, such as dietary composition, enzyme source and activity, application rate and method, rumen environment stability, enzyme-substrate specificity, and the specific dietary component targeted.

Improving feed conversion efficiency, which corresponds to the amount of product consumed per unit of feed, has a good economic and environmental effect on the yak business (9). Feed enzymes are usually added to animal feed to assist in digestion and improve the efficiency of nutrient utilization in the animal digestive system (10). For example, adding a certain concentration of exogenous xylanase to the diet can improve the total tract digestibility of DM, NDF, and ADF in cattle (11). The exogenous enzyme preparations of Aspergillus oryzae and *Aspergillus niger* increased the concentration of milk protein and lactose in cows and increased the milk production of primiparous cows (12). Consequently, incorporating a specific amount of enzyme preparation into yak supplementary feed could enhance forage utilization efficiency, reduce feeding expenses, and play a positive role in improving the ecological environment of the Qinghai Tibet Plateau and enhancing the quality of life of local herdsmen. Nonetheless, there is currently limited available data on the effects of exogenous enzyme preparations on the production performance of yaks. Therefore, the purpose of this work is to analyze the effects of adding ruminant compound enzyme preparations to a diet on feed efficiency, production performance and meat quality in yaks and also attempt to explore the association between changes in gene expression in the liver and meat quality with different concentrations of enzyme preparations in feeding.

## Materials and methods

### Ethics statement

The experimental yaks used in this study were strictly processed by the Animal Ethics Regulations and Guidelines of the People’s Republic of China. This study obtained informed consent from the person in charge of Meiren yak No.2 base. This study has been approved by the Animal Management and Ethics Committee of Lanzhou Institute of Husbandry and Pharmaceutical Sciences, Chinese Academy of Agricultural Sciences (Permit No. 2019–002).

### Animal, diets and experimental design

Thirty healthy adult male yaks (3.5 years old, average weight 279.67 ± 25.37 kg) from the Meiren Yak No. 2 base nucleus herd were randomly divided into three groups (*n* = 10 per group). All yaks were of similar age, weight, and health status, and were maintained under identical feeding and management conditions, except for variations in feed composition. Add different concentrations of ruminant complex enzymes to the basic diet of three groups of yaks, namely LP group (basic diet + 0.05% complex enzymes preparations), MP group (basic diet + 0.10% complex enzymes preparations), and HP group (basic diet + 0.20% complex enzymes preparations). Diets were formulated according to the nutritional requirements for growing finishing beef cattle (275 kg body weight) as per the Feeding Standard of Beef Cattle (NY/T 815–2004). The basal diet composition and nutritional values are detailed in Table 1. The experiment lasted 150 days, including a 15-days adaptation period and a 135-days formal trial. Yaks were fed twice daily (8:00 a.m. and 5:00 p.m.) and provided ad libitum access to water throughout the study.

**Table 1:** The basic dietary composition and nutritional level of yaks (% of DM).

Item	Value
Ingredients	
Corn silage	50.00
Corn	29.00
Cottonseed meal	5.90
Sprouting corn bran	1.50
White Lees	1.50
DDGS	2.55
Bran flour	0.90
Soybean hulls	1.00
Tomato skin	1.00
Soybean meal	2.15
Molasses	1.50
Fine ground limestone	0.75
Salt (NaCl)	0.60
Calcium hydrogen phosphate	0.40
Puffing urea	0.35
Sodium bicarbonate	0.30
Feed grade magnesium oxide	0.10
Ruminant premix	0.50
Chemical composition	
DM	88.98
CP	16.24
ADF	8.20
NDF	16.63

### Measurements and sampling

DMI was calculated based on daily feed provision and residual intake. Each yak was weighed at the beginning and end of the experiment to determine ADG. On the final morning of the experiment, yaks were fasted overnight and slaughtered the following morning (*n* = 4 per group). Immediately after slaughter, samples of the longissimus dorsi muscle, liver tissue, and subcutaneous fat were rapidly collected, snap-frozen in liquid nitrogen, and stored at −80 °C for subsequent fatty acid analysis and RNA extraction. After carcass chilling at 4 °C for 45 min, the longissimus dorsi muscle was sampled between the 12th and 13th ribs on the right side to assess meat color and loin eye area.

### Meat quality

The pH value, meat color, water-holding capacity, and tenderness of the longissimus dorsi muscle were evaluated in slaughtered yaks. Measure the pH values 45 min and 24 h after slaughter, as described by Jin et al. (13). The surface CIE lightness (L*), redness (a*), and yellowness (b*) values of samples were measured by a CR-400 Minolta colorimeter (Konica Minolta Sensing Americas Inc., Ramsey, NJ, USA) with illuminant of D 65 and 8 mm apertures. Calibration was performed prior to color measurement using white plate (Y = 87.0, x = 0.3180, and y = 0.3355) provided by the manufacturer. Each steak was evaluated at three locations on the meat surface. A fresh slice from each sample was weighed (thickness, 30 mm; mean ± SD weight, 150.00 ± 10.00 g), placed in a plastic bag and cooked to an internal temperature of 70 °C in a 75 °C water bath. Next, 50 g of fresh loin was placed in a whirl-Pak bag (Nasco Sampling, Madison, WI, USA), hung in a 4 °C cooler for 48 h and reweighed to determine the drip loss (14). The internal temperature was monitored during cooking with a handheld temperature probe. The cooked samples were cooled for 25 min, blotted dry and weighed. Samples were weighed before and after the test, and the cooking losses were calculated as 100 × (initial weight-final weight)/initial weight. After measuring cooking losses, store the sample at 4 °C for 24 h. The shear force was then measured using a digital tenderness tester (C-LM3B, Tenovo, Beijing, China) the final value calculated as the average of three replicates for each sample.

### Targeted analysis of fatty acid composition in meat

The targeted muscle metabolomics analysis was conducted by Luming Biotechnology in Shanghai, China, utilizing an LC–MS/MS platform. Specifically, approximately 100 mg of frozen samples were ground and homogenized in 600 μL of solution containing 65% methanol and 25% acetone. Firstly, take an ice bath for 20 min, then centrifuge for 10 min. Afterward, dry the supernatant under nitrogen flow and dissolve it in 200 μL of MeOH-2-propanol (1:1, v/v, containing IS). Finally, filter it at 0.22 um and use it for subsequent UPLC-MS/MS analysis. The longissimus dorsi samples were UPLC-ESI-MS/MS analysis following the method of Yu et al. (15). Liquid chromatography and analysis were performed using Nexera UHPLC LC-30A (SHIMADZU) and Waters ACQUITY UPLC BEH C18 columns, respectively. All samples were maintained at a temperature of 4 °C throughout the analysis, while the column temperature was adjusted to 40 °C. Source, operating in negative ion mode; the SCIEXSelex ION Triple Ouad5500 System (Luming Biotechnologyies, Shanghai, China) was used for mass spectrometry. The collision gas used was nitrogen. The following were additional instrumental parameters: curtain gas: 35 Psi; EP:-10 V; IS: 4500 V; CXP:-20 V; TEM: 400C; nebulizer gas: 50Psi; and curtains: 35Sv. MRM mode was used to examine the targeted metabolites and the MRM pairs, respectively, were optimized for each analyst by CE and collision energies. Analyst software was used for data acquisition and additional analysis. Total metabolites were quantified by using the SSCIEX OS-MO software.

### RNA extraction and transcriptome profiling

Total RNA was extracted from the livers of 4 randomly selected yaks from each group. RNA integrity was assessed on Agilent 2,100 BioAnalyzer (Agilent Technologies, Santa Clara, CA, USA). The NEBNext ® Ultra ™ RNA Library Prep Kit for Illumina ® (NEB, Ipswich, MA, USA) was used to construct the RNA library. Sequencing of the library was performed on the Illumina Novaseq 6,000 platform, resulting in paired end reads of 150 bp. After the removal of sequencing adaptors and low-complexity reads the HISAT2 software to map the readings to the reference yak genome assembly BosGru_v3.0 (16). Differential expression analysis was performed using the DESeq2 (17). As shown by the values of the *q* value < 0.05 and the Foldchange ≥ 2, the threshold for the expression of the genes with significant differences was determined. RNA-seq and data analysis commissioned to OE Biotech Co., Ltd. (Shanghai, China). GO enrichment and KEGG pathway enrichment analysis of differently expressed mRNA were, respectively, performed using R based on the hypergeometric distribution (18–20).

### Statistical analysis

Raw data of the meat quality, growth performance, and fatty acid composition were analyzed by one-way ANOVA using SPSS 20.0 software (SPSS Inc., Chicago, IL, USA) to compare the differences between different treatment groups. Calculate the differences between groups using Duncan’s multiple range tests. *p* < 0.05 was considered statistically significant.

## Results

### Growth performance

As shown in Table 2, there were no significant differences in the Initial body weight (IBW) and Final body weight (FBW) among the groups (*p* > 0.05). Simultaneously, compared with the LP group, the average daily weight gain of the HP group was significantly higher than that of the LP group (*p* < 0.05), while the difference between the MP group and the HP group was not significant. On the other hand, there were significant differences in the dry matter intake among the three groups, with the MP group having the highest daily feed intake, followed by the LP group, and finally the HP group (*p* < 0.05). There was a significant difference in feed conversion rate (FCR) among the three groups, with the LP group having the highest FCR, followed by the MP group, and finally the HP group (*p* < 0.05).

**Table 2:** Effects of different diets on the growth performance of yaks.

Parameter	LP	MP	HP	SEM	*P*-value
Initial body weight, kg	282.56	276.00	280.44	2.73	0.97
Final body weight, kg	352.00	358.22	368.33	6.73	0.85
Average daily gain, kg/d	0.63^b^	0.74^ab^	0.82^a^	0.08	0.04
Dry matter intake, kg/d	4.74^b^	4.82^a^	4.66^c^	0.07	0.01
Feed conversion ratio	7.63^a^	6.61^b^	5.48^c^	0.78	0.04

SEM, standard error of the mean (*n* = 10). In the same row, mean values with different superscripts indicate significant differences (*p* < 0.05).

### Meat quality

As shown in Table 3, there were no significant differences in meat quality among different dietary treatments, including pH value, meat color, drip loss, cooking loss, and shear force (*p* > 0.05).

**Table 3:** Effects of different diets on the meat quality of yaks.

Items	LP	MP	HP	SEM	*P*-value
L*	11.32	10.70	11.11	0.26	0.66
a*	23.77	21.70	23.43	0.91	0.17
b*	4.28	3.85	4.42	0.24	0.17
pH45min	6.27	6.14	5.89	0.19	0.12
pH24h	6.06	5.86	5.81	0.09	0.67
Drip loss, %	3.83	3.88	4.02	0.08	0.97
Cooking loss rate, %	24.68	28.05	31.67	2.85	0.60
Shear force, kgf	13.15	12.90	12.96	0.15	0.89

SEM, standard error of the mean (*n* = 4).

### Fatty acid profiles

Table 4 displays the fatty acid levels in the longissimus dorsi muscle of yaks that were fed various diets. A total of 23 metabolites were identified, including 6 short-chain fatty acids and 17 medium-to long-chain fatty acids. Compared with the LP diet, there was no significant difference in the short-, medium-, and long-chain fatty acids between the MP and HP diets statistically.

**Table 4:** The effects of different diets on fatty acid (mg/kg) profiles of the *longissimus dorsi* muscle of yaks.

Fatty acid	LP	MP	HP	SEM	*P*-value
Isovaleric acid (C5:0i)	5.96	29.31	548.98	250.66	0.40
Butyric acid (C4:0)	40.09	194.74	1376.79	597.02	0.36
Acetic acid (C2:0)	8389.67	29087.85	122746.90	49752.73	0.27
Propionic acid (C3:0)	113.52	361.49	4696.83	2104.25	0.41
Isobutyric acid (C4:0i)	13.76	34.72	578.15	261.26	0.41
Pentanoic acid (C5:0)	44.23	106.07	625.35	260.59	0.44
Hexanoic acid (C6:0)	568.34	1355.95	2457.36	774.73	0.47
Palmitic acid (C16:0)	18825.34	15811.25	42133.49	11762.53	0.45
Dodecanoic acid (C12:0)	467.28	719.13	735.21	122.69	0.34
Pentadecanoic acid (C15:0)	149.45	122.77	717.95	274.49	0.40
Margaric acid (C17:0)	264.85	179.15	999.49	368.18	0.42
Stearic acid (C18:0)	11267.56	11459.84	31080.82	9295.07	0.46
Arachidonic acid (C20:4n)	6949.52	2855.71	55526.37	23922.72	0.37
Oleic acid (C18:1n9c)	94.69	34.78	352.87	138.01	0.46
Hexadecenoic acid (C16:1)	180957.52	83906.92	454301.09	156818.14	0.46
Elaidic acid (C18:1n9t)	815.27	431.29	3949.99	1576.04	0.41
Linoleic acid (C18:2n6c)	901.67	595.53	4921.61	1971.14	0.38
Gamma-Linolenic acid (C18:3n6)	35.61	24.99	253.14	105.13	0.39
Linolenic acid (C18:3n3)	837.66	725.21	4302.29	1660.39	0.37
Margaroleic acid (C17:1)	3268.60	1795.60	7173.39	2269.06	0.47
Eicosapentaenoic acid (C20:5n3)	217.26	140.87	1346.43	551.18	0.36
Traumatic acid (C12:2n)	2.05	2.89	6.18	1.78	0.31
Eicosadienoic acid (C20:2n)	285.26	184.01	1159.69	438.03	0.33

### Transcriptome data analysis

**Figure 1:**
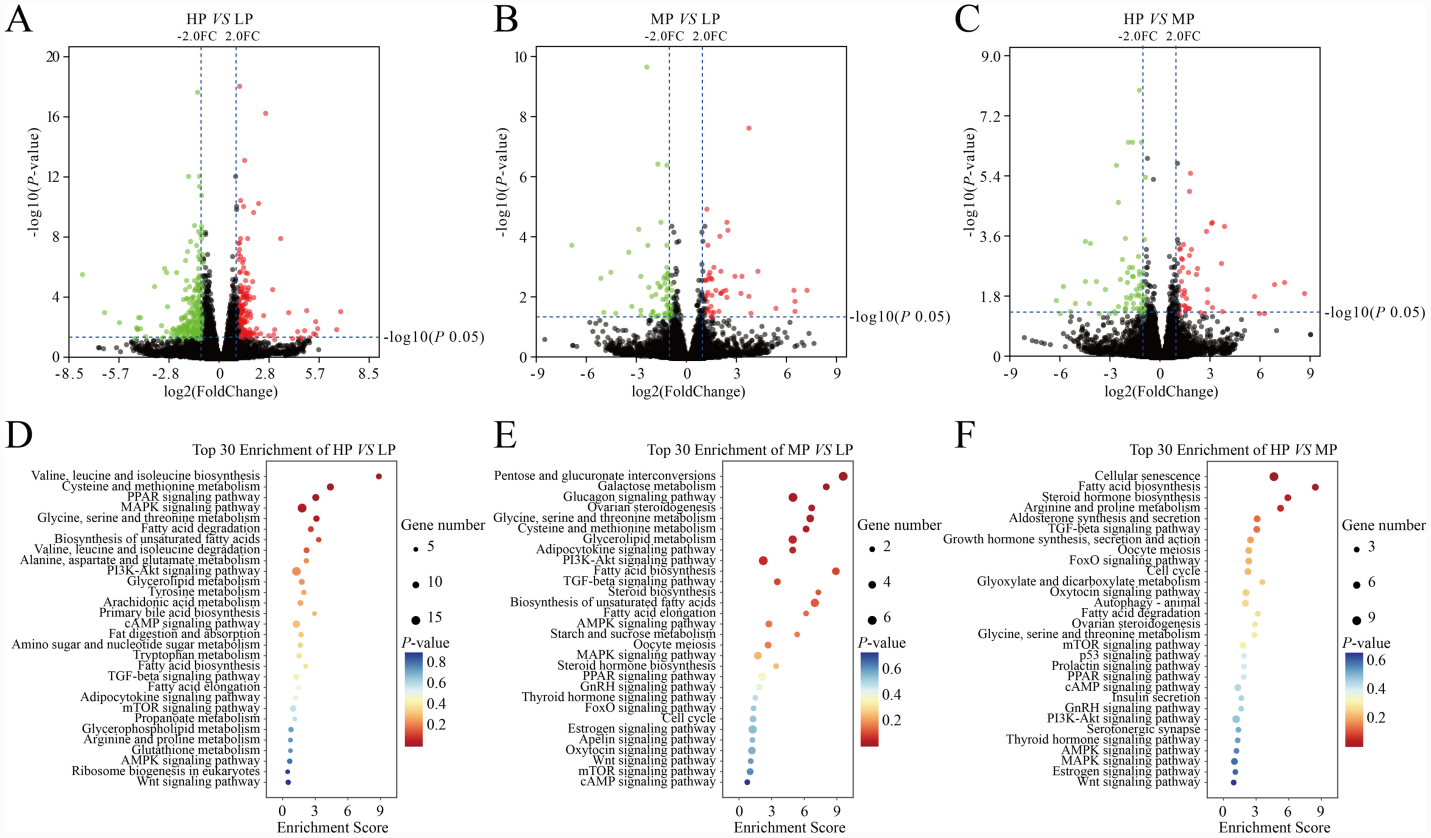
Comparison and functional analysis of mRNA in yak liver treated with different therapies. **(A)** Differences of mRNA expression between HP and LP libraries, **(B)** Differences of mRNA expression between MP and LP libraries, **(C)** differences of mRNA expression between HP and MP libraries, **(D)** KEGG analysis of differentially expressed mRNAs between HP and LP, **(E)** KEGG analysis of differentially expressed mRNAs between MP and LP libraries, **(F)** KEGG analysis of differentially expressed mRNAs between HP and MP libraries. Red, green, and grayness dots in the graph represent transcripts that were significantly up-regulated, down-regulated, and unchanged, respectively.

We used RNA-seq technology to analyzed the differential mRNA expression in three groups of yak liver tissues (LP = 4, MP = 4, HP = 4). The transcriptome results showed that a total of 459 DEGs were identified by comparing the HP group and LP group (Figure 1A). Among them, 159 and 300 genes were up- and down-regulated in the HP group. Comparing the MP group and LP group, a total of 114 DEGs were identified, of which 59 and 64 genes were up- and down-regulated in the MP group (Figure 1B). Comparing the HP group and MP group, a total of 118 DEGs were identified, of which 47 and 71 genes were up- and down-regulated in the HP group (Figure 1C).

In order to better understand the role of differentially expressed mRNA in yak liver, we performed GO and KEGG enrichment analyses on all differentially expressed mRNAs. The GO analysis results showed that the differentially expressed mRNAs were significantly enriched in 171 GO terms between HP and LP, such as organic acid metabolic process, oxidation–reduction process, lipopolysaccharide-mediated signaling pathway, and acetyl-CoA metabolic process. Between MP and LP, a total of 159 significantly enriched GO terms for the differentially expressed mRNAs, in which negative regulation of cell cycle, antigen binding and cell division site were the top GO terms involved in biological processes, molecular functions and cellular components, respectively. Between HP and MP, a total of 183 significantly enriched GO terms for the differentially expressed mRNAs, in which activation of cysteine type endopeptidase activity involved in apoptotic process, transcription factor binding and plasma membrane were the top GO terms involved in biological processes, molecular functions and cellular components, respectively. The KEGG analysis results are shown in Figure 1, as shown in Figures 1D–F, the DEGs in the three comparison groups were involved in PPAR signaling pathway, fatty acid degradation, biosynthesis of unsaturated fatty acids, arachidonic acid metabolism, fat digestion and absorption, fatty acid biosynthesis, and other pathways related to fatty acid metabolism.

## Discussion

In recent years, researchers have collaborated to enhance feed utilization efficiency through the application of exogenous enzymes. Many reports state that the addition of the right amounts of enzyme activity (21, 22) can increase the availability and digestibility of commonly used corn, soybean meal, and other components in livestock diets. Enzyme preparations effectively degrade complex structural components in feed, thereby increasing protein content, soluble carbohydrate levels, and other nutritional parameters while improving feed utilization efficiency (23). Our findings demonstrate that dietary enzyme supplementation significantly enhanced the ADG of fattening yaks, consistent with previous reports by Romero et al. (24). Although the final body weight remained unaffected, the observed improvements in ADG and feed conversion efficiency suggest that the cellulose, hemicellulose and amylase in the compound enzyme preparation likely enhanced nutrient digestibility, absorption, and utilization, ultimately improving yak fattening performance (25). Meat quality represents one of the most economically important characteristics in yak production. Key meat quality parameters include tenderness (measured by shear force), water-holding capacity, pH, and color (26). In the present study, increasing concentrations of compound enzyme preparations in feed did not significantly affect drip loss, cooking loss, or shear force. Post-mortem pH decline, a critical indicator of meat quality that influences shelf life and storage duration (27), results from accelerated muscle glycolysis and subsequent lactic acid accumulation (28). Our results showed no significant differences in pH decline rates between 45 min and 24 h post-slaughter across treatment groups, indicating that enzyme supplementation did not affect yak meat spoilage rates or shelf life. Meat color, a primary visual determinant of consumer preference, is typically assessed using L* (lightness), a* (redness), and b* (yellowness) values, with higher a* and lower L* values generally indicating superior quality (29). However, dietary enzyme supplementation in this study did not significantly influence these color parameters in yak meat.

The fatty acid composition of muscle tissue serves as a critical determinant of both meat quality attributes and potential health benefits for consumers (30). As Alvarenga et al. have demonstrated, the balance between saturated (SFA) and polyunsaturated fatty acids (PUFA) in meat products has significant implications for human cardiovascular health, with particular benefits associated with increased PUFA intake (31). While our results showed that the high-concentration enzyme preparation (HP) group exhibited numerically higher total fatty acid content in muscle tissue compared to other groups, it is noteworthy that these differences did not reach statistical significance. This observation suggests that while enzyme supplementation may influence fatty acid deposition patterns, the effects may be subtle or require longer intervention periods to become statistically detectable. Of particular nutritional interest were the trends observed for two key unsaturated fatty acids, Oleic acid is a monounsaturated fatty acid, known for its beneficial effects on blood lipid profiles and cholesterol metabolism (32), and showed a modest increase in the 0.20% enzyme supplementation group. Linoleic acid as an essential PUFA with demonstrated cardio protective properties (33), its elevated levels in enzyme-supplemented groups, though not statistically significant, may still have important implications for meat quality. These patterns, while not statistically significant in our study, align with the growing body of evidence suggesting that dietary interventions can modulate muscle fatty acid profiles in ruminants. The observed trends toward increased unsaturated fatty acid content in enzyme-supplemented groups may reflect the enhanced ruminal biohydrogenation escape of dietary unsaturated fats, modified hepatic lipid metabolism and export patterns and altered muscle lipid uptake and incorporation mechanisms. From a practical perspective, even modest increases in beneficial fatty acids like oleic and linoleic acid could improve the nutritional value of yak meat products. However, the lack of statistical significance in our findings suggests that the higher supplementation levels may be needed to achieve significant effects, longer feeding durations might be required to observe measurable changes and individual variation in yak metabolism may require larger sample sizes. These results have important implications for yak production systems aiming to enhance the health promoting properties of meat while maintaining traditional feeding practices. Future research should investigate the optimal enzyme supplementation protocols for maximizing beneficial fatty acids, potential interactions between enzyme preparations and other dietary components and the stability of these fatty acid modifications during meat processing and storage. While our current findings show promising trends rather than statistically significant changes, they nevertheless contribute to our understanding of how dietary enzyme supplementation might be utilized to improve the nutritional quality of yak meat products for health conscious consumers.

The liver serves as the central metabolic hub for lipid homeostasis, orchestrating the storage, metabolism, and redistribution of fatty acids in ruminants (34). Our transcriptome analysis of yak liver tissues revealed significant alterations in gene expression profiles in response to varying concentrations of enzyme preparations, providing novel insights into the molecular mechanisms underlying dietary enzyme-induced modifications of fatty acid metabolism. The dose-dependent response observed in our study was particularly noteworthy. While moderate enzyme supplementation (MP group) resulted in relatively modest changes (50 up-regulated and 64 down-regulated genes compared to LP), high-concentration supplementation (HP group) induced more profound transcriptional reprogramming, with 159 genes up-regulated and 300 down-regulated relative to LP. This graded response suggests that enzyme preparations may modulate hepatic metabolism through multiple regulatory thresholds, with higher concentrations potentially activating additional metabolic pathways. Of particular significance was the enrichment of differentially expressed genes (DEGs) in the PPAR signaling pathway. As PPARα serves as the dominant isoform in ruminant liver and a master regulator of fatty acid oxidation and transport (35), its activation may represent a central mechanism through which enzyme preparations influence lipid metabolism. The coordinated changes observed in downstream pathways including fatty acid degradation, unsaturated fatty acid biosynthesis, and arachidonic acid metabolism suggested comprehensive remodeling of hepatic lipid handling.

The medium-chain acyl-CoA dehydrogenase (ACADM) serves as the critical initiator of mitochondrial *β*-oxidation for medium-chain fatty acids (C6-C12) (36). Our observed alterations in ACADM expression patterns suggest that enzyme supplementation may modify hepatic energy derivation from lipids. This finding is particularly significant given that ACADM deficiency is well-documented to cause profound disruptions in fatty acid catabolism and hepatic function (37). The modulation of ACADM expression by dietary enzymes could therefore represent a fundamental mechanism influencing overall energy metabolism in yaks. Apolipoprotein A5 (APOA5), predominantly synthesized in hepatocytes (38), emerged as another crucial regulator. As the most potent genetic modulator of triglyceride metabolism identified to date (39), APOA5 likely mediates the crosstalk between hepatic lipid processing and peripheral fat deposition. The differential expression of APOA5 observed in our study suggests that enzyme preparations may alter the interorgan distribution of fatty acids, potentially affecting muscle lipid composition through modified lipoprotein metabolism. The mitochondrial enzyme 3-hydroxy-3-methylglutaryl-CoA synthase 2 (HMGCS2), which catalyzes the rate-limiting step in ketogenesis (40), showed significant expression changes across treatment groups. As the primary determinant of hepatic ketone body production, HMGCS2 plays a dual role in maintaining intrahepatic lipid homeostasis while coordinating whole-body energy distribution during different metabolic states (41). The observed transcriptional changes in HMGCS2 may reflect adaptive responses to altered nutrient availability resulting from enzyme supplementation, potentially influencing the balance between lipid storage and utilization in peripheral tissues. The identification of other lipid metabolism regulators (PLIN5, ME1, and ACOX1) further strengthens the hypothesis that enzyme preparations induce a coordinated transcriptional response affecting multiple nodes of hepatic lipid metabolism. These findings raise important questions about the potential for dietary enzyme supplementation to influence meat quality through modulation of fatty acid profiles. Our findings provide a molecular foundation for understanding how enzyme preparations may improve the nutritional quality of yak products through modulation of hepatic lipid metabolism. The identification of these potential regulatory genes and pathways offers new targets for optimizing yak feeding strategies and enhancing the health-promoting properties of yak meat.

## Conclusion

In summary, our findings demonstrate that dietary supplementation with 0.20% enzyme pre-rations significantly improves growth performance in yaks, evidenced by the increasing average daily weight gain, reducing feed intake, and improving feed conversion efficiency. However, meat quality parameters (including pH, water-holding capacity, and color stability) remained unaffected by enzyme supplementation. Notably, transcriptomic analysis revealed significant alterations in the expression of hepatic genes involved in fatty acid metabolism pathways, suggesting that enzyme preparations may modulate lipid metabolism at the molecular level. These results indicate that while enzyme supplementation offers clear benefits for yak growth performance, its effects on meat quality and hepatic metabolic pathways require further investigation. Further research is needed to elucidate the mechanisms linking hepatic gene expression changes to growth performance, determine optimal supplementation strategies and evaluate long-term effects on both animal health and product quality. This study provides valuable insights for developing enzyme assisted feeding regimens in yak production systems, while highlighting important avenues for future investigation.

## Data Availability

The data presented in the study are deposited in the Sequence Read Archive repository, accession number PRJNA1414832.
